# Anticipatory and consummatory pleasure in eating disorders

**DOI:** 10.1186/s40337-022-00692-w

**Published:** 2022-11-12

**Authors:** Sarah C. Dolan, Erin E. Reilly, Tiffany A. Brown, Megan E. Shott, Guido K. W. Frank

**Affiliations:** 1 Department of Psychology, Hofstra University, Hempstead, USA; 2 Department of Psychiatry, University of California, San Francisco, USA; 3 Department of Psychology, Auburn University, Auburn, USA; 4 Department of Psychiatry, University of California San Diego, 4510 Executive Drive, San Diego, CA 92121 USA

**Keywords:** Eating disorders, Binge eating, Pleasure, Anhedonia

## Abstract

**Background:**

Recent research suggests that anhedonia, or the inability to experience pleasure, is elevated in individuals with eating disorders (EDs). However, past literature has only studied anhedonia in EDs as a unidimensional construct rather than separately examining anticipatory (i.e., prediction of pleasure for a future event) and consummatory (i.e., enjoyment of a present event) pleasure. Given that these subcomponents of pleasure have distinct neurobiological correlates, studying pleasure as a multifaceted construct may yield important insights into the underlying mechanisms of binge eating or food restriction.

**Methods:**

A sample of 124 women with anorexia nervosa, bulimia nervosa, or other specified feeding or eating disorder and 84 control women (CW) completed self-report measures of anticipatory pleasure, consummatory pleasure, ED symptoms, depression, harm avoidance, and anxiety.

**Results:**

Individuals with EDs endorsed significantly lower anticipatory pleasure than CW, but there were no significant group differences in consummatory pleasure. Further, there were no significant differences in self-reported pleasure among ED diagnostic groups. Within the ED sample, anticipatory pleasure but not consummatory pleasure was positively related to binge eating frequency and significantly negatively correlated with cognitive ED symptoms, state and trait anxiety, and harm avoidance. Both anticipatory and consummatory pleasure was negatively associated with depression.

**Conclusion:**

The results of the current study suggest that lower pleasure across the ED spectrum may be due to deficits in anticipatory, but not consummatory, pleasure. Future research should continue to explore the behavioral, affective, and neural correlates of anticipatory pleasure in EDs to characterize better how it relates to the onset and maintenance of binge eating and other eating disorder pathology.

## Plain English summary

Anhedonia, or the inability to experience pleasure, has been observed in individuals with eating disorders. Neuroscience research suggests that pleasure may be separated into two distinct components: anticipatory pleasure (how much someone predicts they will enjoy a future experience) and consummatory pleasure (how much someone enjoys a present experience). In the current study, individuals with eating disorders and healthy controls completed questionnaires assessing anticipatory and consummatory pleasure, binge eating, other eating disorder behaviors, depression, anxiety, and constructs associated with reward and punishment sensitivity. The sample with eating disorders reported significantly lower anticipatory but not consummatory pleasure than the control sample. Within the eating disorder sample, greater anticipatory pleasure was also related to higher binge eating frequency but lower depression, anxiety, and weight and shape concerns. These results suggest that anticipatory pleasure may be particularly important in future research on the etiology and treatment of eating disorders.

## Background

A range of theoretical models has implicated alterations in reward processing in the etiology and maintenance of eating disorders (EDs) [1–4]. Much of this work was initially driven by observations that individuals with EDs often endorse increased anhedonia [5, 6] outside the context of depression, alongside altered activity in reward-related brain regions to disorder-specific stimuli (i.e., thinness, weight loss, food) [7]. In addition, recent theoretical work outside of EDs has proposed that because anhedonia appears to be driven by neurobiological alterations in the reward system [8, 9], which also appears to be altered in EDs, treatments that target reward-related symptoms and anhedonia may help improve long-term outcomes [10, 11]. However, the field’s knowledge regarding specific aspects of hedonic processing has been limited by (a) a lack of consideration of different subcomponents of reward processing that may influence an individual’s ability to experience pleasure and (b) insufficient consideration of anhedonia across a range of ED diagnoses. Further identifying how hedonic processing is altered in different ED diagnostic profiles (e.g., binge eating spectrum) will aid in formulating effective, targeted treatments. Therefore, in the current study, we were seeking to extend existing work in anhedonia and EDs by exploring subcomponents of the ability to experience pleasure—anticipatory (prediction regarding whether an experience will be gratifying) and consummatory (whether the experience is gratifying once received)—in a mixed diagnosis sample of individuals with binge eating-spectrum EDs, restricting-type EDs, and healthy control individuals.

Anhedonia is traditionally considered a central symptom of depression [12, 13] but is observed transdiagnostically and appears to be related to alterations in the positive valence system and reward processing [14]. Regarding its role in EDs, as noted above, a growing body of literature suggests that EDs are generally characterized by elevations in anhedonia or lack of enjoyment of pleasurable stimuli [15], although findings have been mixed. A recent meta-analysis suggests that anhedonia appears elevated across ED diagnoses even after accounting for depressive symptoms [15]. Further, while some preliminary work indicates that anhedonia may decrease relative to baseline over the course of ED treatment, individuals with EDs continue to report clinically-significant elevations in anhedonia at discharge and follow-up compared to healthy control individuals [11, 16]. To date, investigations are limited by a primary focus on anorexia nervosa and other restricting-type EDs, despite initial findings supporting elevated anhedonia across other ED diagnoses and significant alterations in reward processing in binge eating-spectrum disorders [17]. Therefore, further work is needed to characterize anhedonia and reward processing more comprehensively across the spectrum of ED diagnostic categories.

In addition to a relative neglect of binge eating-spectrum EDs in research on pleasure and reward processing, the existing literature on this topic is also limited by the predominant consideration of pleasure as a unidimensional construct. Outside of EDs, research in reward processing suggests that reward processing is characterized by a number of subcomponents with distinct neurobiological substrates. While there exist several theoretical frameworks for understanding components of reward processing, one early and commonly-cited model suggested that reward processing may include (a) “liking,” or consummatory pleasure, (b) “wanting,” or motivation to pursue rewards, and (c) “learning,” or incorporating information regarding the receipt of rewards into future behaviors and predictions about reward [18, 19]. More recent theoretical models of reward, such as that of the Research Domain Criteria (RDoC), posit that reward processing can be further separated into as many as nine subconstructs, including the addition of reward satiation and subconstructs of reward learning and reward valuation [20]. Because there are likely a number of distinct processes within the reward circuitry that could contribute to anhedonia and lack of pleasure, symptoms of anhedonia can manifest across one or more of these areas, such as the decreased ability to experience motivation to pursue rewards or decreased subjective pleasure when consuming rewards. Within the current study, we consider both anticipatory pleasure, which refers to an individual’s prediction of whether or not and to what degree an individual expects an experience to be gratifying, as well as consummatory pleasure, which refers to whether or not the receipt of the reward is gratifying [21].

Altogether, the investigation of subcomponents of reward processing and pleasure in EDs will inform novel interventions and future research on the role of reward in the etiology and maintenance of eating pathology. Specifically, identifying how subconstructs of the ability to experience pleasure may differentially relate to ED symptoms will refine reward-based theoretical models of ED and influence future directions for research in this area. In addition, elucidating the roles of specific components of reward and pleasure may ultimately contribute to developing novel interventions that target these areas, as has been proposed in other disorders [22].

### Current study

The current study had two central aims. First, we aimed to build on prior research documenting elevated anhedonia in EDs by exploring differences in mean levels of anticipatory and consummatory pleasure in a mixed diagnostic sample of patients with anorexia nervosa (AN) bulimia nervosa (BN), other-specified eating disorder (OSFED), and healthy control women (CW), with a particular focus on comparing anticipatory and consummatory pleasure between binge eating-spectrum EDs with primarily restricting-type EDs and CW. Based on a past meta-analysis suggesting elevations in anhedonia in ED samples but few diagnostic differences [15], we anticipated that the ED groups would demonstrate decreased consummatory and anticipatory pleasure; however, there would be no differences across diagnostic categories. Our second aim was to probe associations between anticipatory and consummatory pleasure with relevant psychiatric symptoms, including binge eating and other ED symptoms, depression, and anxiety. As self-reported anticipatory and consummatory pleasure has not been evaluated in EDs to date, this aim was exploratory, and we did not have any a priori hypotheses.

## Methods

### Participants & procedure

Participants for the present study were adult women with EDs (n = 124) and healthy control women (CW; n = 84) who completed self-report measures as part of a larger study on the neurobiology of reward and eating [23] (see Table 1 for demographics). Participants with EDs were recruited from two ED partial hospitalization programs within the first two weeks of treatment. The diagnostic makeup of the ED group was as follows: 51 (41.13%) AN, 40 (32.26%) BN, and 33 (26.61%) OSFED. CW were recruited from the local community via flyers and had no history of psychiatric or major medical illness, including no history of an ED. All participants were right-handed without history of head trauma, neurological disease, major medical illness, bipolar disorder, psychosis, or current (past three months) substance use disorder. Both ED and CW participated between March 2014 and June 2019. ED participants were more likely to self-identify as white, were younger, and had fewer years of education.

**Table 1:** Diagnostic and clinical characteristics of the sample

	AN (n = 51)	BN (n = 40)	OSFED (n = 33)	CW (n = 83)	*F/χ*^2^*	*p*
	M(SD)/n(%)	M(SD)/n(%)	M(SD)/n(%)	M(SD)/n(%)		
Race						
White	47 (92.2)	35 (87.5)	31 (93.9)	66 (78.6)	14.33	0.006
Asian/Pacific Islander	1 (2.0)	1 (2.5)	1 (3.0)	12 (14.3)		
Black/African American	1 (2.0)	3 (7.5)	1 (3.0)	3 (3.6)		
More than one race	1 (2.0)	1 (2.5)	0	2 (2.4)		
Declined to answer	1 (2.0)	0	0	0		
Hispanic/latinx	4 (7.8)	0	1 (3.0)	7 (8.3)	1.67	0.20
Age	22.1 (5.4)	23.1 (4.0)	22.3 (6.0)	25.5 (3.4)	7.80	< 0.001
Years of education	12.9 (3.5)	13.6(3.6)	12.0 (3.2)	16.6 (2.4)	25.99	< 0.001
Body mass index	15.9 (1.2)	23.2 (7.9)	20.3 (3.6)	N/A		
BDI	30.0 (12.6)	30.2 (11.1)	32.3 (11.9)	1.8 (2.3)	161.08	< 0.001
TEPS-anticipatory	3.5 (0.8)	3.8 (0.9)	3.4 (0.9)	4.5 (0.6)	55.68	< 0.001
TEPS-consummatory	4.9 (0.8)	5.0 (0.9)	4.8 (0.9)	5.1 (0.6)	2.69	0.44
State anxiety	55.7 (11.6)	58.0 (11.9)	58.5 (12.9)	26.0 (6.7)	153.98	< 0.001
Trait anxiety	57.7 (11.7)	62.1 (11.4)	59.4 (11.4)	27.2 (5.8)	189.57	< 0.001
Harm avoidance	21.8 (8.0)	24.2 (7.1)	23.9 (7.0)	11.0 (5.4)	55.89	< 0.001
EDE-Q global	3.2 (1.3)	4.0 (1.1)	3.9 (0.9)	0.4 (0.4)	207.09	< 0.001
Restraint	3.4 (1.9)	3. (1.6)	4.1 (1.)	0.6 (0.8)	83.30	< 0.001
Eating concern	3.1 (1.4)	3.9 (1.3)	3.8 (1.3)	0.1 (0.2)	189.87	< 0.001
Shape concern	4.4 (1.7)	5.0 (1.5)	5.2 (1.1)	0.7 (0.8)	187.56	< 0.001
Weight concern	3.9 (1.8)	4.6 (1.5)	5.0 (1.2)	0.5 (0.6)	168.63	< 0.001
Binge frequency	1.1 (2.2)	3.0 (2.0)	0.4 (1.0)	0.04 (0.2)	37.21	< 0.001
Vomiting frequency	1.2 (2.2)	3.6 (2.5)	2.2 (2.8)	0 (0)	35.84	< 0.001
Laxative frequency	0.3 (0.6)	1.4 (2.2)	0.5 (1.3)	0 (0)	14.15	< 0.001
Exercise frequency	2.7 (2.6)	1.9 (2.2)	2.3 (2.1)	0.2 (0.8)	21.90	< 0.001

*BDI* Beck depression inventory; *CW* Control women; *ED* Eating disorder; *TEPS* Temporal experience of pleasure scale, *EDE-Q* Eating disorder examination questionnaire

*Tests of group differences were performed between the combined ED sample and control sample

Written informed consent was obtained before participation, and the local Institutional Review Board approved all study procedures. As part of eligibility screening, all participants were assessed with the Structured Clinical Interview for DSM-5 Axis I Disorders (SCID) [24]. The SCID was conducted by a doctoral-level interviewer trained in the assessment, the principal investigator, or a staff psychologist. The study team reviewed diagnoses. Eighty-five (68.5%) of ED participants were diagnosed with a current mood disorder, 98 (79.0%) were diagnosed with a current anxiety disorder, 22 (17.7%) were diagnosed with obsessive-compulsive disorder, and 44 (35.5%) were diagnosed with posttraumatic stress disorder. Among the individuals with comorbid anxiety disorders, the most common diagnoses were GAD (n = 61) and social phobia (n = 44). Seventy-three ED participants (58.9%) were prescribed antidepressants, 17 (13.7%) were prescribed an atypical anti-psychotic, and 14 (11.3%) were prescribed mood stabilizers.

### Measures

*Pleasure* was measured using the Temporal Experience of Pleasure Scale (TEPS) [25]. The TEPS is an 18-item scale that assesses the experience of pleasure using a total score and two subscales: anticipatory pleasure and consummatory pleasure. Lower scores indicate anhedonia, and higher scores indicate greater anticipatory and consummatory pleasure. Cronbach’s alpha in the present study was adequate for the TEPS total score (α = 0.82), anticipatory subscale (α = 0.77), and consummatory subscale (α = 0.75).

*Depression* was assessed using the Beck Depression Inventory-II (BDI) [26]. The BDI is a 21-item, well-validated self-report questionnaire used to evaluate the severity of depressive symptoms. Internal consistency within the present sample was α = 0.91.

*ED symptoms* were assessed using the Eating Disorder Examination Questionnaire (EDE-Q) [27]. The EDE-Q is a 28-item self-report measure that assesses ED symptom severity within the past 28 days. The EDE-Q has four primary subscales–shape concern, weight concern, dietary restriction, and eating concern—which are scored using a 7-point (0–6) scale, with higher scores indicating greater severity. To assess the frequency of binge eating episodes and compensatory behaviors (self-induced vomiting, laxative use, and excessive exercise), which are not included in any of the subscales, participants report the number of days on which these behaviors occurred in the past 28 days.

*Anxiety* was assessed using the State-Trait Anxiety Inventory (STAI) [28], a 40-item self-report questionnaire that measures state anxiety (i.e., how anxious one feels at the present moment) and trait anxiety.

*Harm avoidance* was measured using the harm avoidance subscale of the Temperament and Character Inventory [29]. This 35-item self-report measure assesses fear of uncertainty, shyness around others, fatiguability, and anticipatory worry about future harmful events.

### Statistical analyses

Data were examined and determined not to be normally distributed. As such, nonparametric tests were used for primary analyses. To address Aim 1, Kruskal-Wallis tests were run comparing CW and ED diagnoses (AN, BN, and OSFED) on TEPS anticipatory and consummatory subscales. Significant tests were followed up with Bonferroni-corrected pairwise comparisons. To address Aim 2, exploratory Spearman correlations were also run, comparing associations between the TEPS subscales, BDI, state and trait anxiety, and harm avoidance within the ED sample. To further explore relationships between pleasure and ED symptoms, we calculated Spearman correlation coefficients among TEPS subscales, EDE-Q subscales, and frequency of binge episodes and compensatory behaviors within the ED sample. Using the recommendations of Cohen [30], a correlation coefficient of ± 0.1 was considered small, ± 0.3 was considered moderate, and ± 0.5 was considered large.

To assess for the potential confounding effects of demographic and clinical variables on TEPS subscales, significant tests were followed up with an ANCOVA with group (AN, BN, OSFED or control) as the primary independent variable of interest; major depressive disorder diagnosis, race, age, and years of education entered as covariates, and TEPS score as the dependent variable. Data were rank transformed.

## Results

Demographics and descriptive characteristics of study variables for both the ED and control samples are presented in Table 1.

### Group differences in anticipatory pleasure

There were significant group differences on the TEPS anticipatory subscale across individuals with AN, BN, OSFED, and CW. The overall test was significant, *H*(3) = 55.68, *p* < .001, and follow-up pairwise comparisons indicated that, after correcting for multiple comparisons, the CW group reported greater anticipatory pleasure than individuals with AN (*p* < .001), BN (*p* < .001), and OSFED (*p* < .001). Pairwise comparisons did not indicate that any of the ED diagnoses significantly differed from each other on anticipatory pleasure.

The ANCOVA model that included depression, race, years of education, and age, comparing group differences in anticipatory pleasure was significant, *F*(7, 199) = 12.08, *p* < .001. There was a significant effect of group, *F*(1, 199) = 4.81, *p* = .003, and major depressive disorder diagnosis, *F*(7, 199) = 5.10, *p* = .025 on anticipatory pleasure, such that individuals with ED diagnoses or major depressive disorder endorsed lower anticipatory pleasure compared to controls and individuals without comorbid depression. Race, years of education, and age did not significantly affect anticipatory pleasure. Post hoc comparison indicated lower anticipatory pleasure in AN (p = .001), BN (p = .032), and OSFED (p = .001) groups compared to CW.

### Group differences in consummatory pleasure

There were no significant differences in the TEPS consummatory scores across individuals with AN, BN, OSFED, and CW, *H*(3) = 2.69, *p* = .44, suggesting that individuals with EDs experience similar levels of consummatory pleasure to those without any history of psychiatric illness.

### Correlates of TEPS scores in EDs

All Spearman correlation coefficients from these analyses are available in Table 2. The TEPS subscales significantly and moderately correlated with each other, *r = .*40, *p* < .001, suggesting that individuals who experience greater anticipatory pleasure also experience greater consummatory pleasure. Both anticipatory and consummatory pleasure was moderately negatively related to depression, *r = −* .37, *p* < .001, and *r = *− .19, *p* = .03, respectively. Of note, although lower anticipatory pleasure was moderately related to higher state anxiety (*r = −* .31, *p* < .001), trait anxiety (*r = −* .24, *p* = .01), and harm avoidance (*r = −* .31, *p* = .01), consummatory pleasure did not have a significant relationship with these variables.

**Table 2:** Spearman correlation coefficients of main study variables in the eating disorder group

	BDI	TEPS-C	TEPS-A	STAI-S	STAI-T
HA	0.56*	− 0.13	− 0.31*	0.49	0.59*
STAI-T	0.73**	− 0.13	− 0.24*	0.77**	
STAI-S	0.57**	− 0.11	− 0.31**		
TEPS-A	− 0.37**	0.40**			
TEPS-C	− 0.19*				

*BDI* Beck depression inventory, *HA* Harm avoidance, *STAI-S* State-trait anxiety inventory state subscale, *STAI-T* State-trait anxiety inventory trait subscale, *TEPS-A* Temporal experience of pleasure scale anticipatory subscale, *TEPS-C* Temporal experience of pleasure scale consummatory subscale

*p < .05

**p < .01

The full results of correlation analyses assessing relationships between TEPS scores and EDE-Q subscales are available in Table 3. Anticipatory pleasure was significantly negatively related to eating concern (*r = −* .23, *p* = .01), shape concern (*r = −* .24, *p* = .01), weight concern (*r = −* .21, *p* = .02), and excessive exercise (*r = −* .21, *p* = .02), suggesting that individuals who experience greater anticipatory pleasure have lower symptom severity in these domains. Anticipatory pleasure was positively associated with binge frequency (*r =* .21, *p* = .02), suggesting that more frequent binge eating episodes were associated with higher self-reported anticipatory pleasure. Anticipatory pleasure did not significantly correlate with self-induced vomiting or laxative use. Notably, consummatory pleasure had no significant relationship with any ED symptoms assessed by the EDE-Q.

**Table 3:** Spearman correlation coefficients of pleasure and eating disorder symptoms as assessed by the Eating Disorder Examination Questionnaire in the eating disorder group

	DR	EC	SC	WC	Binge	Vomiting	Laxative	Exercise
TEPS-A	*−* 0.12	*−* 0.23*	*−* 0.24**	*−* 0.21*	0.21*	0.13	*−* 0.05	*−* 0.21*
TEPS-C	0.12	0.02	0.01	0.02	0.12	*−* 0.04	*−* 0.01	0.01

*TEPS-A* Temporal experience of pleasure scale anticipatory subscale, *TEPS-C* Temporal experience of pleasure scale consummatory subscale; *DR* Dietary restraint; *EC* Eating concern; *SC* Shape concern; *WC* Weight concern

*p < .05

**p < .01

## Discussion

The present study extends prior work on anhedonia to compare self-reported anticipatory and consummatory pleasure among individuals with AN, BN, and OSFED and its links with clinical symptoms, filling an important gap in a body of literature that has previously examined anhedonia and pleasure as a unidimensional construct with a primary focus on anorexia nervosa.

Based on meta-analytic findings that indicate elevated anhedonia in samples with EDs [15], we hypothesized that the ED group would endorse lower levels of consummatory and anticipatory pleasure compared to controls. However, individuals with EDs only reported decreased anticipatory pleasure, suggesting that future research on anhedonia in EDs should continue to assess multiple subtypes of pleasure to identify better how individuals with EDs may experience deficits in pleasure. These findings are consistent with some evidence from behavioral tasks that have found that ED symptoms are associated with decreased “wanting” (i.e., anticipatory pleasure) but not “liking” (i.e., consummatory pleasure) when presented with rewarding stimuli [31]. This difference in subtypes of pleasure provides additional detail to results from previous literature that suggests samples with EDs report increased overall anhedonia compared to control groups [10, 32]; however, past studies have not directly assessed subtypes of anhedonia or pleasure. As such, it is unknown whether the group differences reported in these studies may have been due to differences in anticipatory pleasure but not consummatory pleasure. Importantly, though early research on anhedonia in EDs was focused mainly on anorexia nervosa [33], we found that ED diagnostic groups endorsed similar levels of pleasure, indicating that low anticipatory pleasure may be relevant to EDs associated with restrictive or binge eating behaviors. Recently, we found in a brain imaging study investigating expectation and receipt of a caloric taste stimulus elevated anxiety and altered amygdala response across EDs to expectation but not receipt of the stimulus [34]. These two studies are consistent as they emphasize negative emotionality to expectation but a normal response to the experience of the stimulus.

Overall, correlations among study variables in the ED sample are consistent with previous research in clinical and community samples, suggesting that the TEPS is negatively correlated with depression symptoms [35] and positively related to measures of reward sensitivity [36]. Because these results show a similar pattern of relationships between self-report measures of pleasure and reward responsiveness across different clinical samples, this initial data suggests that models of reward processing and anhedonia tested in other clinical populations (e.g., depression) may have relevance for the study of reward in EDs. Although both anticipatory and consummatory pleasure had similar patterns of relationships among study variables, anticipatory but not consummatory pleasure was significantly negatively related to anxiety and harm avoidance, suggesting that deficits in anticipatory pleasure in EDs may intersect with observed elevations in harm and punishment avoidance [37]. For instance, individuals with low anticipatory pleasure and increased state or trait anxiety may be more likely to perceive future stimuli as dangerous rather than potentially enjoyable. In fact, anticipatory anxiety and perhaps anxious traits may lead to anhedonia related to anticipatory pleasure. However, these variables were only moderately correlated, indicating that other factors likely also contribute to self-reported deficits in anticipatory pleasure. Because the current study provides preliminary evidence that depression, anxiety, and ED symptoms relate to decreased anticipatory pleasure in EDs, future research should identify which psychiatric symptom domains most strongly relate to loss of pleasure in this population.

Concerning relationships between pleasure experiences and specific ED symptoms, anticipatory pleasure was negatively related to a number of ED cognitive symptom categories (weight, shape, and eating concerns). However, the directionality of this relationship is unclear—individuals with EDs may have premorbid low anticipatory pleasure, which makes them less likely to pursue rewarding activities outside of ED-relevant behaviors, or increasing ED symptom severity may contribute to blunted anticipatory pleasure.

In contrast to the negative relationships between anticipatory pleasure and cognitive ED symptom domains, this construct was positively related to binge eating frequency. The positive relationship between anticipatory pleasure and binge eating may occur due to expectancies that binge eating will alleviate negative affect or increase positive affect, which has been observed in individuals who engage in binge eating and purging behaviors [38, 39]. Alternately, individuals with binge eating symptoms may expect that eating will increase positive affect [40]; future research should explore relationships between anticipatory pleasure and eating expectancies in samples with binge eating. Importantly, the current study only assessed binge eating frequency; future research on anticipatory pleasure and binge eating should also probe relationships between cognitive and affective constructs associated with binge eating (such as shame or disgust) [13] to characterize better how dimensions of pleasure relate to binge eating episodes.

Notably, consummatory pleasure in the ED sample was neither significantly different from CW nor related to any ED symptom categories, indicating that the study of anticipatory pleasure specifically may be an important direction for future research on reward in EDs. In addition, anticipatory pleasure may be particularly relevant to observed differences in decision-making in individuals with EDs [41, 42], such that individuals with EDs may not anticipate typically rewarding stimuli to be enjoyable and worth pursuing.

The current study is novel in assessing multiple subcomponents of pleasure, but it has several limitations. First, although both TEPS subscales demonstrated adequate internal consistency in this sample, this measure has not been validated in samples with EDs. To ensure that the TEPS is an accurate measure of pleasure in this population, future studies should compare this questionnaire with other measures of anhedonia and pleasure more commonly used in samples with EDs, such as the Snaith-Hamilton Pleasure Scale [43] and the Chapman anhedonia scales [44]. Additionally, while the TEPS measures dimensions of pleasure in a general sense, the current study did not include a measure that specifically probed anticipatory or consummatory pleasure relating to food (e.g., Power of Food Scale) [45] which is clinically relevant in the study of pleasure and reward in EDs. Finally, our sample represented a wide range of ED diagnoses, but no participants were diagnosed with binge eating disorder. Although there were no significant differences in anticipatory or consummatory pleasure within ED diagnostic groups, it is unknown whether individuals with binge eating disorder may endorse differing levels of pleasure. Furthermore, a follow-up ANCOVA found that major depressive disorder was related to significantly lower anticipatory pleasure in addition to an ED diagnosis. This is consistent with prior research indicating that depression is linked to decreased anticipatory and consummatory pleasure [21] and meta-analytic findings that suggest that comorbid depression symptoms may contribute to anhedonia in individuals with EDs [15]; it is important for future research in this area to identify whether and how depression and ED symptoms may differentially relate to low pleasure in this population.

Additionally, the methods of the current study were based upon early multidimensional models of reward [19] that focus on three primary subcomponents (“liking,” “wanting,” and “learning”) because these models are conceptually consistent with the TEPS, which measures anticipatory (“wanting”) and consummatory (“liking”) pleasure. However, as noted earlier, more recent research efforts (e.g., RDoC) have suggested that reward may have substantially more distinct subcomponents [20]; future research examining dimensions of pleasure in EDs should seek to incorporate a more comprehensive model of reward processing.

In keeping with some neurobiologically-informed models of reward suggesting that reward processing includes both anticipatory and consummatory phases [19], the results of this study indicate the need for future research on anhedonia and pleasure in EDs to study these constructs as multifaceted rather than unidimensional. In particular, studies should examine self-report measures of anhedonia or pleasure alongside reward-related neuroimaging and behavioral paradigms to more clearly delineate how aspects of subjective experiences of pleasure relate to observed neurocognitive differences in reward processing [46]. Future research should also assess whether this pattern of decreased anticipatory but not consummatory pleasure can be extended to disorder-specific domains, such as food and appearance-related stimuli.

In sum, the current study offers promising preliminary data suggesting that deficits in pleasure in ED samples may be due to anticipatory but not consummatory anhedonia. Furthermore, these findings give insight into how subcomponents of pleasure may differentially relate to ED symptoms and provide a compelling rationale for the future study of how self-reported anhedonia may relate to neurobiological and behavioral indices of reward processing in EDs.

## Data Availability

The data used in the current study are available on request from the corresponding author.

## Abbreviations

EDs: Eating disorders
AN: Anorexia nervosa
BN: Bulimia nervosa
OSFED: Other specified feeding or eating disorder
CW: Control women
RDoC: Research domain criteria
SCID: Structured clinical interview for DSM-5 axis I disorders
GAD: Generalized anxiety disorder
TEPS: Temporal experience of pleasure scale
BDI: Beck depression inventory-II
EDE-Q: Eating disorder examination questionnaire
SPSRQ: Sensitivity to punishment and sensitivity to reward questionnaire
STAI: State-trait anxiety inventory
